# Prevalence and characteristics of malaria among COVID-19 individuals: A systematic review, meta-analysis, and analysis of case reports

**DOI:** 10.1371/journal.pntd.0009766

**Published:** 2021-10-01

**Authors:** Polrat Wilairatana, Frederick Ramirez Masangkay, Kwuntida Uthaisar Kotepui, Giovanni De Jesus Milanez, Manas Kotepui

**Affiliations:** 1 Department of Clinical Tropical Medicine, Faculty of Tropical Medicine, Mahidol University, Bangkok, Thailand; 2 Department of Medical Technology, Institute of Arts and Sciences, Far Eastern University-Manila, Manila, Philippines; 3 Medical Technology, School of Allied Health Sciences, Walailak University, Tha Sala, Nakhon Si Thammarat, Thailand; 4 Department of Medical Technology, Faculty of Pharmacy, University of Santo Tomas, Manila, Philippines; University of Florida, UNITED STATES

## Abstract

**Background:**

The world population is currently at a very high risk of Coronavirus disease-2019 (COVID-19), caused by the severe acute respiratory syndrome-coronavirus-2 (SARS-CoV-2). People who live in malaria-endemic areas and get infected by SARS-CoV-2 may be at increased risk of severe COVID-19 or unfavorable disease outcomes if they ignore their malaria status. Therefore, the present study aimed to synthesize, qualitatively and quantitatively, information on the prevalence and characteristics of malaria infection among COVID-19-infected individuals. The findings will help us better understand this particular comorbidity during the COVID-19 pandemic.

**Methods:**

The systematic review protocol was registered at the International Prospective Register of Systematic Reviews (PROSPERO) with the identification number: CRD42021247521. We searched for studies reporting on the coinfection of COVID-19 and malaria in PubMed, Web of Science, and Scopus from inception to March 27, 2021 using Medical Subject Headings (MeSH) terms. The study’s methodological quality in the search output was assessed using the Joanna Briggs Institute (JBI) Critical Appraisal Tools for cross-sectional study. The pooled prevalence of *Plasmodium* spp. infection among patients infected with COVID-19 was estimated using the random effect model and then graphically presented as forest plots. The heterogeneity among the included studies was assessed using Cochrane Q and I^2^ statistics. The characteristics of patients co-infected with COVID-19 and malaria were derived from case reports and series and were formally analyzed using simple statistics.

**Results:**

Twelve of 1,207 studies reporting the coinfection of COVID-19 and malaria were selected for further analysis. Results of quantitative synthesis show that the pooled prevalence of *Plasmodium* spp. infection (364 cases) among COVID–19 individuals (1,126 cases) is 11%, with a high degree of heterogeneity (95% CI: 4%–18%, I^2^: 97.07%, 5 studies). Most of the coinfections were reported in Nigeria (336 cases), India (27 cases), and the Democratic Republic of Congo (1 case). Results of qualitative synthesis indicate that patients with coinfection are typically symptomatic at presentation with mild or moderate parasitemia. An analysis of case reports and series indicates that co-infected individuals often display thrombocytopenia, lymphopenia, and elevated bilirubin levels. Among four patients (30%) who required treatment with intravenous artesunate, one experienced worsened clinical status after administering the drug. One serious outcome of coinfection involved a pregnant woman who experienced fetal abortion due to the initial misdiagnosis of malaria.

**Conclusions:**

All individuals in malaria-endemic regions who are febrile or display symptoms of COVID-19 should be evaluated for malaria to avoid serious complications. Further prospective studies are required to investigate the burden and outcomes of COVID-19 in malaria-endemic regions. Prompt management is required to prevent serious outcomes in individuals co-infected with COVID-19 and malaria.

## Author summary

The world population is currently at a high risk of Coronavirus disease-2019 (COVID-19), which is caused by severe acute respiratory syndrome-coronavirus-2 (SARS-CoV-2). People who live in malaria-endemic areas and get infected by SARS-CoV-2 may be at an increased risk of severe COVID-19 if they are co-infected with malaria. Therefore, we aim to demonstrate the prevalence and characteristics of malaria infections among COVID-19-infected individuals. From the systematic literature review, we showed that the prevalence of *Plasmodium* spp. infection among patients with COVID–19 is 11%. Most of the coinfections were reported in Nigeria, India, and the Democratic Republic of Congo. Moreover, we showed that patients with coinfections are typically symptomatic at presentation with mild or moderate parasitemia. We also indicated that patients with coinfections often display thrombocytopenia, lymphopenia, and elevated bilirubin levels. Among four patients with coinfections and severe diseases, one experienced worsened clinical status after administration of the anti-malarial drug. One of the patients, a pregnant woman, experienced a serious outcome of coinfection, fetal abortion, due to the initial misdiagnosis of malaria. Based on our study, further prospective studies must investigate the burden and outcomes of COVID-19 in malaria-endemic regions. Further prompt management is necessary to prevent severe outcomes in patients co-infected with COVID-19 and malaria.

## Background

Severe acute respiratory syndrome-coronavirus 2 (SARS-CoV-2) infection causes the novel coronavirus disease (COVID-19). It is currently affecting the world’s entire population. According to the most recent report of the World Health Organization (WHO), there are more than 142 million confirmed cases and 3 million deaths due to this disease globally [1]. The spread of COVID-19 in Africa has been slower than its spread in other areas such as the Americas, Europe, South-East Asia, and countries in the Eastern Mediterranean. Nevertheless, 3 million cases and over 78,000 deaths due to COVID-19 have been recorded in Africa [1]. The relatively low spread of COVID-19 in Africa may depend on the host’s genetic epidemiology and other related factors that protect the African population from SARS-CoV-2 infection [2]. The pathogenesis of SARS-CoV-2 infection in humans involves ACE2, a virus receptor, and the S protein, a serine protease involved in priming the viral spike (S) protein [3,4]. In the respiratory tract, SARS-CoV-2 binds to and replicates in the alveolar epithelial cells of the lungs. The replication of SARS-CoV-2 in the lungs may trigger a strong immune response at the sites of infection, causing mild symptoms, acute respiratory distress syndrome (ARDS), respiratory failure, or death in high-risk patients [5].

Globally, 229 million malaria cases and 409,000 deaths were estimated in 2019 by WHO [6]. African countries account for approximately 94% of malaria cases and deaths worldwide, whereas about 3% were reported by WHO in South-East Asia regions such as India, Indonesia, Bangladesh, Nepal, Thailand, Sri Lanka, Myanmar, and Maldives [6]. In major endemic areas like Africa, malaria healthcare interventions and control measures, such as chemoprevention and long-lasting insecticide treatment of bed nets against mosquitoes, are likely to be limited during the COVID-19 pandemic due to lockdown [7]. Therefore, COVID-19 affects the efforts of healthcare providers in controlling malaria, which eventually leads to increase in malaria incidence. This increase resulting from the overlap of two pathogens, is a cause of great concern in malaria-endemic areas due to overlapping geographical pathogens causing the COVID-19 and malaria coinfections. However, there is a significant knowledge gap concerning the coinfection of these two diseases. A better understanding of coinfection may result in the development of control strategies for co-endemic areas. Hence, we aimed to synthesize, qualitatively and quantitatively, the current knowledge on the pooled prevalence and characteristics of patients co-infected with COVID-19 and malaria to better understand malaria as a comorbidity of COVID-19.

## Methods

### Protocol and registration

The systematic review protocol was registered at the International Prospective Register of Systematic Reviews (PROSPERO) under identification number CRD42021247521. The review follows the preferred reporting items for systematic reviews and meta-analyses (S1 PRISMA Checklist) [8].

### Search strategy

We searched for all potentially relevant studies that reported on the coinfection of COVID-19 and malaria in the PubMed, Web of Science, and Scopus databases, focusing on articles published from inception to 27 March 2021. We used the following search phrase: (“COVID-19” OR “2019-nCoV” OR “2019 nCoV Infection” OR “2019-nCoV” OR “Coronavirus Disease-19” OR “Coronavirus Disease 19” OR “2019 Novel Coronavirus” OR COVID19 OR “COVID 19” OR “COVID-19” OR “SARS Coronavirus 2” OR “SARS-CoV-2”) AND (malaria OR Plasmodium). Only terms included in the Medical Subject Headings (MeSH) were used in the search strategy (S1 Table).

### Eligibility criteria

We included only original articles, case reports, and case series published in English that reported on the coinfection of COVID-19 and malaria in the present study. The study excluded review articles, commentaries/corrections/news/letters to the editor/short reports/communications, in vitro studies, articles on COVID-19 and non-relevant parameters, COVID-19 model predictions, molecular docking studies, non-relevant case reports, systematic reviews or scoping reviews, community implementation studies, surveys, clinical trials, animal studies, and cost-analytic studies.

### Study selection and data extraction

During study selection by two authors (MK, PW), any initial disagreement regarding eligibility was resolved by discussion among all the authors and then agreement by consensus. The following data were extracted from each study for further analysis: first author, year of publication, study location, the year the study was conducted, participants’ gender and age, number of patients with coinfection, number of patients infected with SARS-CoV-2 only, and number of patients infected with *Plasmodium* spp. were extracted into a standardized pilot datasheet before further analysis.

### Quality of the included studies

The quality of this study was assessed independently by two authors (MK, PW) according to the Joanna Briggs Institute (JBI) Critical Appraisal Tools for cross-sectional study [9]. Disaggrements between the two authors were resolved by consensus with the help of a third reviewer (FRM). Following are the Key aspects of JBI Critical Appraisal Tools: 1) defined criteria for inclusion of study participants, 2) the description of study participants and the setting, 3) validity and reliability of exposure measurement, 4) objective and standard criteria used for measurement of the condition, 5) identification of confounding factors, 6) strategies to deal with confounding factors, 7) validity and reliability of the outcome measurement, and 8) appropriate statistical analysis. For the quality assessment, “High,” “Moderate,” or “Low,” quality was rated for any study given more than 7 scores, 4−6 scores, and <4 scores, respectively.

### Statistical analysis

The pooled prevalence of *Plasmodium* spp. infection among the included studies was estimated using the random effect models (DerSimonian and Laird). The forest plots show the results of individual and pooled analyses of included studies. The heterogeneity of the study results among the included studies was assessed using Cochrane Q and I^2^ statistics. The Cochrane Q statistic was considered significant at p < 0.05, while an I^2^ value of greater than 50 indicated substantial heterogeneity. If substantial heterogeneity was detected, then the random effect models were used to pool the effect measure. If no significant heterogeneity was detected, then the fixed effect models were used to pool the effect measure. The publication bias was assessed visually using a funnel plot and Egger’s test for asymmetry if the number of included studies was more than 10. The data synthesis was performed using STATA Statistical Software version 15.0 (StataCorp. 2017. Stata Statistical Software: Release 15. College Station, TX: StataCorp LLC). The characteristics of patients co-infected with SARS-CoV-2 and *Plasmodium* spp. were analyzed using simple statistics such as frequency and percentages. Results of analysis of case reports and series are presented as frequencies or graphs.

## Results

### Search results

**Fig 1:**
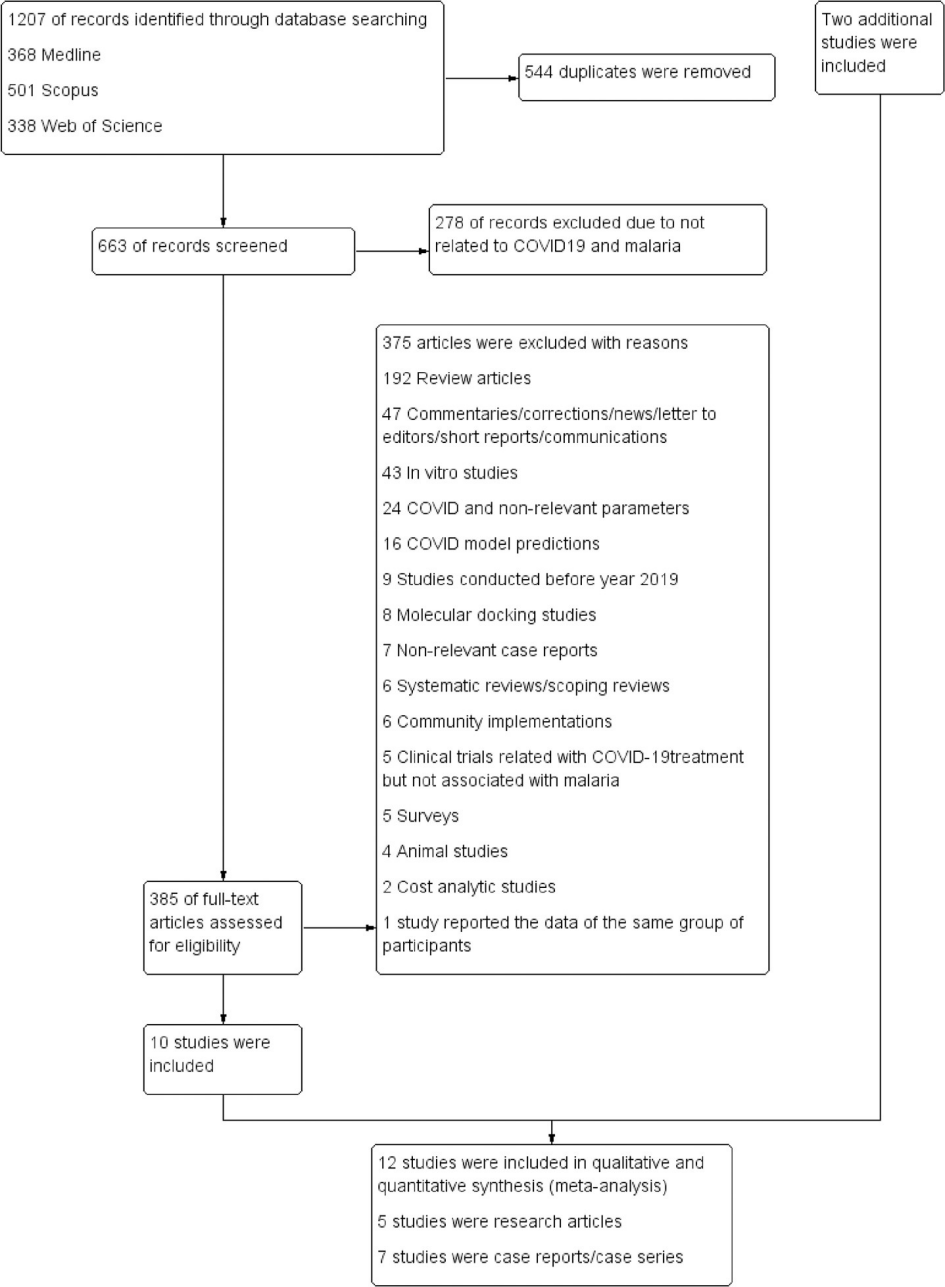
Flowchart for the selection of potentially relevant studies for use in the present study.

Our database searches yielded 1,207 articles, comprising 368, 501, and 338 articles from PubMed, Scopus, and Web of Science, respectively. After removing all duplicates, the titles and abstracts of the remaining 663 articles were screened, resulting in excluding 278 articles because they were unrelated. We examined the full-texts of the remaining 385 articles, resulting in the exclusion of 375 articles due to the following reasons: 192 were review articles; 47 were commentaries, corrections, news items, letters to the editor, short reports, and communications; 43 were in vitro studies; 24 were about COVID-19 only, with non-relevant parameters; 16 were COVID-19 model predictions; 9 were conducted before 2019; 8 were molecular docking studies; 7 were non-relevant case reports; 6 were systematic and scoping reviews; 6 were community implementations; 5 were surveys; 5 were clinical trials related to COVID-19 treatment efficacy/safety but not associated with malaria; 4 were animal studies; 2 were cost-analytic studies; and 1 study reported the data of the same group of participants. After these exclusions, 10 studies [10–19] met the eligibility criteria and were included. An additional two studies [20,21] were retrieved from Google Scholar and included. Thus, a final set of 12 studies [10–21] were included for qualitative and quantitative synthesis (meta-analysis) and analysis of co-infected cases. Among the 12 studies included, 5 [10–13,21] were original research articles, and 7 [14–20] were case reports or series (Fig 1).

### Characteristics of research articles

Among the five included studies, four were cross-sectional studies [11–13,21], and one was a retrospective cohort study [10]. Three studies were conducted in Nigeria [11,12,21], one in India [13], and one was conducted in the Democratic Republic of Congo [10]. Four studies enrolled patients in tertiary city hospitals, while one study enrolled patients at the COVID-19 Drive-through testing center. Three studies [10–12] (3/4, 75%) enrolled individuals with confirmed COVID-19, while two studies enrolled individuals with suspected COVID-19 [13,21]. The diagnosis of COVID-19 relied on RT-PCR by four studies [10,11,13,21], whereas one study did not specify the laboratory confirmation of COVID-19 [12]. The diagnosis of malaria among three studies relied on microscopy [10–12], one used microscopy, or RDT [13], and one study used only RDT [21]. All studies conducted malaria diagnosis during COVID-19 due to investigations on possible correlations between malaria and COVID-19 [21], the prevalence of malaria infection among COVID-19 [10], the risk of oxidative stress in the coinfection of malaria and COVID-19 [11], the role of sex in the coinfection of malaria and COVID-19 [12], and the impact of malaria coinfection on the viral clearance in patients with COVID–19 [13] (Table 1). Clinical characteristics of patients with COVID-19 are shown in Table 2. Only two studies [10,13] demonstrated the clinical severity of COVID-19, treatment of COVID-19, and mean duration during hospitalization. Three studies demonstrated underlying comorbidities [10,13,21]. Most of the patients with COVID-19 in two studies [10, 13] were of mild severity, treated with hydroxychloroquine or chloroquine phosphate, and had comorbidity mostly with hypertension and diabetes.

**Table 1:** Characteristics of the included studies.

No.	Author, year	Study design	Study location (year)	Type of health facility	Participants enrolled (n)	Gender	Age (years)	No. of patients with COVID19	COVID19 diagnosis	Malaria diagnosis	Do all patients with COVID-19 had blood smears performed?	How was it decided to do a smear on the COVID patients?
1.	Amoo *et al*., 2020 [21]	Cross- sectional study	Nigeria (2020)	COVID-19 Drive-through testing center	Patients suspected with COVID-19 (617)	Male (358) Female (259)	Mean 38 ± 11	121	RT-PCR	RDT	Yes	To explore the possible correlation between malaria and COVID-19
2.	Matangila *et al*., 2020 [10]	Retrospective cohort study	Democratic Republic of Congo	Tertiary city hospital	Patients with COVID-19 (160)	Male (82), female (78)	Mrdian 54 years (IQR: 38–64)	160	RT-PCR	Microscopy	Yes	To explore the prevalence of malaria infection among COVID-19
3.	Muhammad *et al*., 2020 [11]	Cross- sectional study	Nigeria (2020)	Tertiary city hospital	Participants (74): patients with COVID-19 (54), healthy controls (20)	NS	NS	54	RT-PCR	Microscopy	Yes	To explore the risk of oxidative stress in the coinfection of malaria and COVID-19
4.	Onosakponome *et al*., 2020 [12]	Cross- sectional study	Nigeria	Tertiary city hospital	Patients with COVID-19 (300)	Male (179), female (121)	NS	300	NS	Microscopy	Yes	To explore the role of sex in the coinfection of malaria and COVID-19
5.	Mahajan et *al*., 2021 [13]	Cross- sectional study	India (2020)	Tertiary city hospital	Front-line health-care workers (3,711)	COVID19 (491): male (267), female (224)	COVID19 (491): median 32 years (27–44.5)	491	RT-PCR	Microscopy or RDT	Yes	To explore the impact of malaria coinfection on the virus clearance in patients with COVID-19

NS: Not specified

**Table 2:** Clinical characteristics of COVID-19.

No.	Author, year	No. of COVID19	No. of malaria as a coinfection	*Plasmodium* spp.	Clinical severity of COVID19	Treatment for COVID-19	Mean duration of hospitalized (mean days and SD)	Underlying comorbidities
1.	Amoo *et al*., 2020 [21]	121	2	*P*. *falciparum*	Asymtomatic (52%), mild (48%)	NS	NS	Heart diseases (7%), asthma (3%),hypertension (3%), pregnancy (3%), obesity (2%), chronic liver disease (2%), chronic kidney disease (1%), chronic lung disease (1%), anemia or chronic hematology (1%)
2.	Matangila *et al*., 2020 [10]	160	1	*Plasmodium* spp.	Mild (57%), moderate (12%), severe (31%)	Hydroxychloroquine or chloroquine phosphate (92%)	15 (4–20)	Hypertension (34%), diabetes (19%), obesity (8%), heart disease (7%), asthma/ chronic pulmonary disease (3%)
3.	Muhammad *et al*., 2020 [11]	54	34	*Plasmodium* spp.	NS	NS	NS	NS
4.	Onosakponome *et al*., 2020 [12]	300	300	*P*. *falciparum*	NS	NS	NS	NS
5.	Mahajan et *al*., 2021 [13]	491	27	*P*. *vivax*	Mild (73.9%), moderate (12.5%), severe (2.4%)	Hydroxychloroquine (53.9%)	21 (14–35)	Hypertension (11%), diabetes (8%), bronchial asthma (4%), hypothyroidism (2.8%), tuberculosis (1.1%), ischemic heart disease (1.3%), other comorbidity (1.7%), more than 1 comorbidity (6.5%)

NS: Not specified

**Fig 2:**
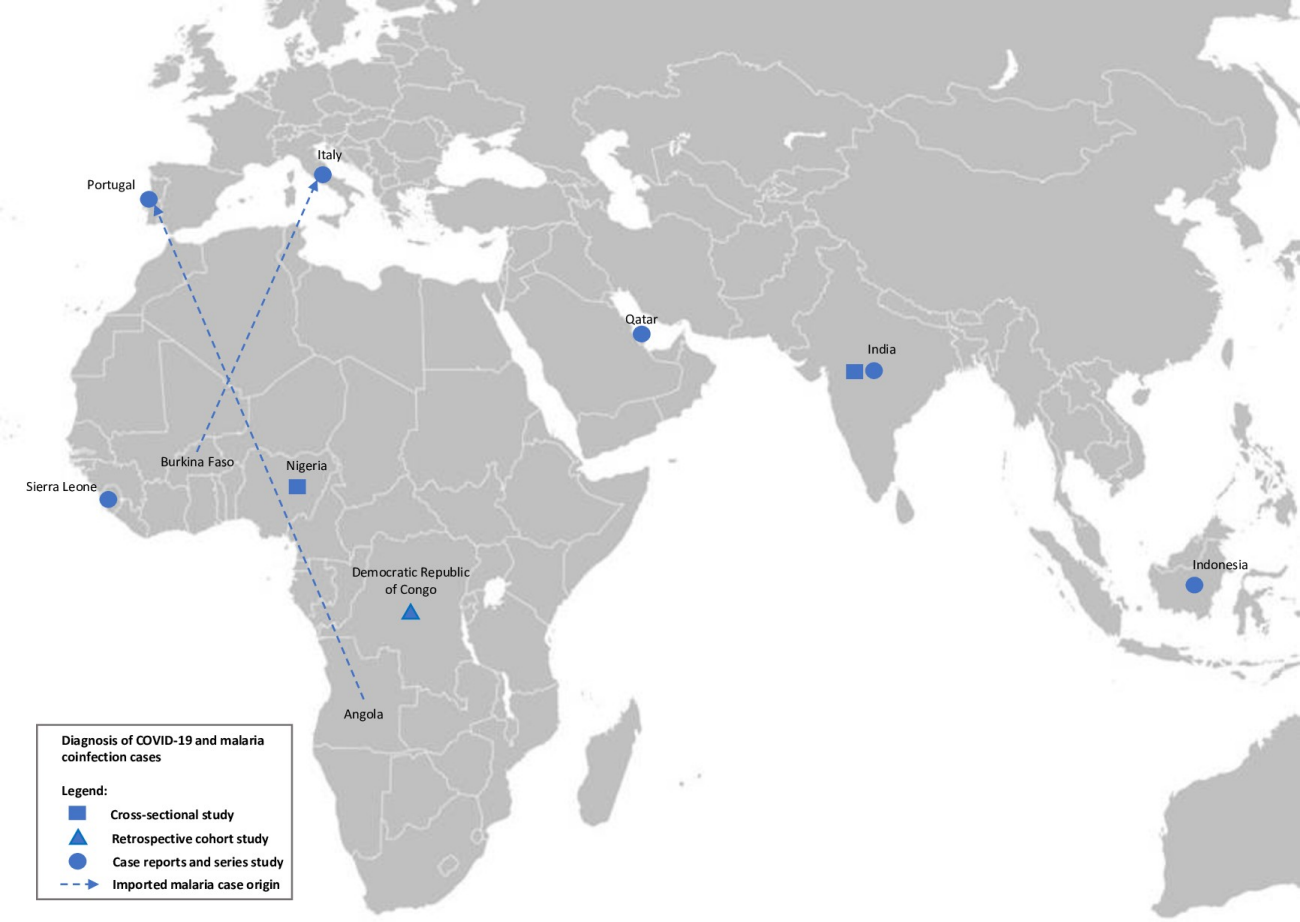
Global distribution of *Plasmodium* spp. infection among patients with COVID-19 (blue color). The map was created from the map template available at https://commons.wikimedia.org/wiki/Atlas_of_the_world.

Seven case reports and series reported a total of 12 patients with coinfections; 4 patients were from India [17,20], 4 were from Sierra Leone [14], and the rest were from Portugal [15], Indonesia [16], Qatar [18], and Italy [19] (Fig 2). Additionally, imported malaria was reported in patients who traveled to Angola [15] and Burkina Faso [19]. The ages of patients with coinfection ranged from 4−67 years (median age 29.5 years). *Plasmodium falciparum* infections were reported by two studies [15,16], whereas *P*. *vivax* infections were reported by two studies [17,20]. Most patients with coinfection were females (8/12, 66.7%) (S1 Data).

### Quality of the included studies and publication bias

The quality of the studies was assessed according to the JBI Critical Appraisal Tools for a cross-sectional study. Four studies [10,12,13,21] were high-quality studies, whereas only one [11] was moderate-quality study (S2 Table). We were unable to assess publication bias because less than 10 studies were original articles [22].

### Prevalence of *Plasmodium* spp. infection among patients with COVID-19

**Fig 3:**
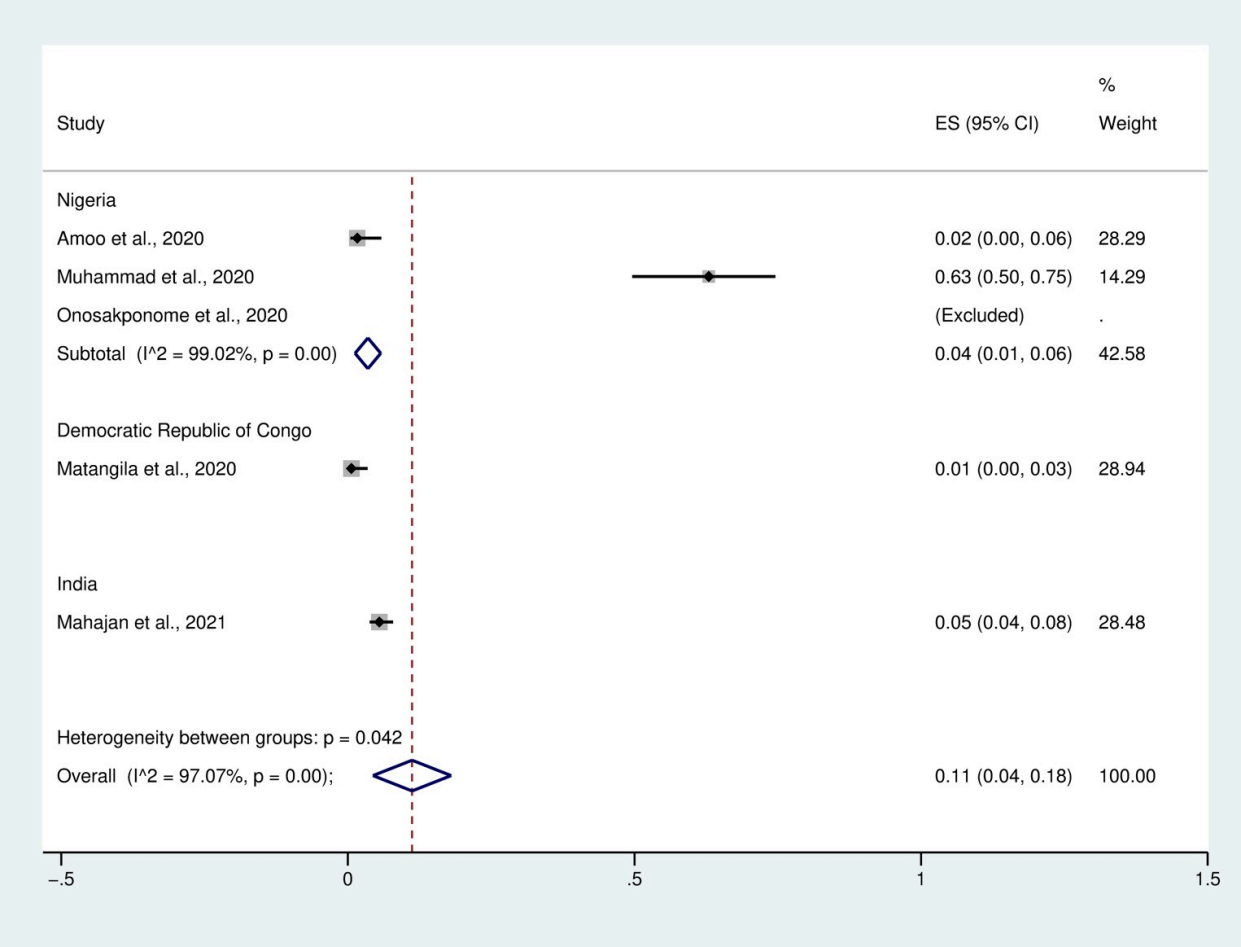
Forest plot of the pooled prevalence of *Plasmodium* spp. infection among patients with COVID-19. ES, effect size; CI, confidence interval.

Estimation of the pooled prevalence of *Plasmodium* spp. infections among patients with COVID-19 (coinfection) from data of five studies [10–13,21] yielded an estimate of 11% pooled prevalence with a high degree of heterogeneity (95% CI: 4%–18%, I^2^: 97.07%, 364/1,126 cases) (Fig 3). The subgroup analysis based on region demonstrated that the pooled prevalence of *Plasmodium* spp. infection among patients with COVID-19 in India is 5% (95% CI: 4%–8%, 27/491 cases) while that in the Democratic Republic of Congo is 1% (95% CI: 0%–3%, 1/160 cases) and that in Nigeria is 4% (95% CI: 1%–6%, I^2^: 99.02%, 336/475 cases). Notably, a study by Onosakponome *et al*. [12] in Nigeria reported a 100% rate of *Plasmodium* spp. infection among patients with COVID-19 (100% coinfection) enrolled in their study.

**Fig 4:**
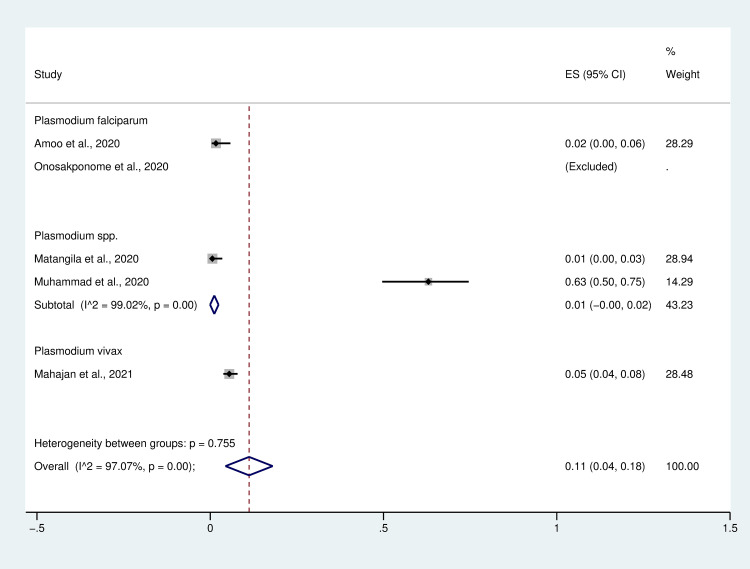
Forest plot of the subgroup analysis of the pooled prevalence of *Plasmodium* spp. infection among patients with COVID-19. ES, effect size; CI, confidence interval.

The subgroup analysis based on *Plasmodium* spp. demonstrated that the pooled prevalence of *Plasmodium* spp. infections (species not reported by studies) among patients with COVID-19 is 1% (95% CI: 0%–2%, 35/214 cases), whereas *P*. *vivax* and *P*. *falciparum* infections among patients with COVID-19 is 5% (95% CI: 4%–8%, 27/491 cases) and 2% (95% CI: 0%–6%, 2/121 cases). Notably, a study by Onosakponome *et al*. [12] in Nigeria reported a 100% rate of *Plasmodium falciparum* infection among patients with COVID-19 (100% coinfection) enrolled in their study (Fig 4).

### Signs or symptoms at presentation

For patients infected with COVID-19, Amoo *et al*. [10] reported that most patients upon admission were asymptomatic (52%), whereas the remaining patients had mild diseases (48%), including fever (29%), sore throat (24%), and dry cough (20%). Matangila *et al*. reported that most of the symptoms reported upon admission were mild (57%), and among those including fever (58%), cough (57%), fatigue (54%), and shortness of breath (45%). Additionally, Mahajan *et al*. [13] also reported that the most common symptoms were mild (73.9%), among those including fever (64%), dry cough (29%), myalgia (27%), and sore throat (24%). Among the five original articles included in the present study, four [10,12,13,21] reported on the clinical signs or symptoms of patients with coinfection at first presentation. Amoo *et al*. [21] reported that all patients with coinfection in their study were asymptomatic (2 cases), while Onosakponome *et al*. [12] reported that most of the patients with coinfection had fever (79.7%) upon presentation, while some had coughs (17.7%), difficulty breathing (7%), headaches (19%), sore throats (4.67%), and runny noses (11%). Matangila *et al*. [10] also reported on patients with coinfection and severe clinical presentations, but the exact nature of the complications was not described. While Mahajan *et al*. [23] described the prevalence of coinfection, they did not report on the signs or symptoms at presentation. However, 85% of the healthcare workers investigated in their study were symptomatic. Also, Mahajan *et al*. [13] reported that patients with coinfection had a shorter duration of viral clearance or days of recovery (7 days, IQR 6–8 days) than those with COVID–19 coinfection alone (11 days, IQR 7–15 days).

Clinical presentations of patients with coinfection were well-described in case reports and case series. Among the 12 cases of coinfection described in seven case reports and series, the study by Adetola et al. [14] reported that three out of four cases of coinfection were asymptomatic at presentation; the remaining one case presented with cough. Mahajan et al. [17] reported that two out of three cases of coinfection were asymptomatic at presentation, with the one remaining case presenting with influenza-like symptoms. Five case reports [15,16,18–20] describe five cases of coinfection, all of which presented with fever and non-specific symptoms. Overall, out of all 12 cases of coinfection, 41.7% were asymptomatic, while the rest (58.3%) presented with fever and non-specific signs or symptoms such as chills, diarrhae, malaise, diaphoresis, occasional dry cough, vomiting, abdominal pain, jaundice, and progressive exertional breathlessness.

### Malaria parasitemia

Among five original articles included in the present study, two [10,12] described the levels of malaria parasitemia among cases of coinfection. Matangila *et al*. [12] reported a coinfection with a parasite density of 16,900/μL [10]. Meanwhile, Onosakponome et al. reported that 19.3%, 27.7%, and 53% of cases of coinfection involved high, moderate, and low levels of parasitemia, respectively.

Out of 12 cases of coinfection described in seven case reports and series, three [15,16,18] reported on malaria parasitemia among patients with coinfection. Correia *et al*. [15] reported a parasite level of 83,520 parasites/μL (3.1% parasitemia), whereas Junaedi *et al*. [16] and Sardar *et al*. [18] reported parasite levels of 119,902 parasites/μL and 1.2% parasitemia, respectively.

### Laboratory parameters

**Fig 5:**
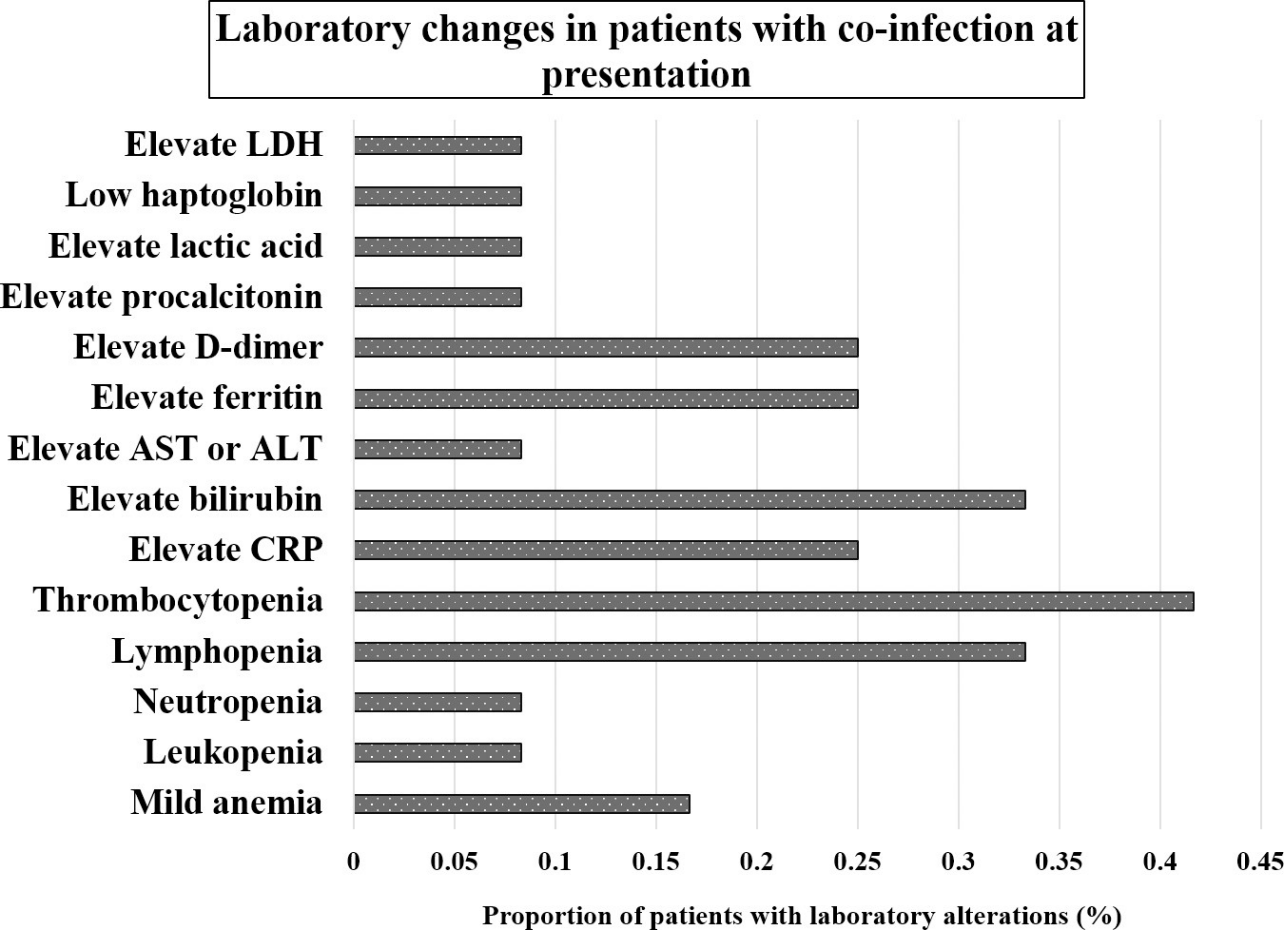
Laboratory alterations of patients with coinfection at presentation.

For COVID-19 monoinfected patients described in the main study, most of the patients had normal leukocyte count (72.1%), higher CRP (100%), higher procalcitonin (74%), higher D-dimer (67%), elevated AST (66%), elevated ALT (37%), nearly normal urea, and creatinine [10]. Alterations of laboratory parameters in patients with coinfection were described in seven case reports and series [14–20]. Out of 12 patients with coinfection, thrombocytopenia (41.7%, 5/12), lymphopenia (33.3%, 4/12), elevated bilirubin (33.3%, 4/12), elevated D-dimer (25%, 3/12), elevated ferritin (25%, 3/12), elevated CRP (25%, 3/12), and mild anemia (16.7, 2/12) were common laboratory alterations reported in patients with coinfection, whereas elevated LDH (8.3%, 1/12), low haptoglobin (8.3%, 1/12), elevated lactic acid (8.3%, 1/12), elevated procalcitonin (8.3%, 1/12), elevated AST or ALT (8.3%, 1/12), neutropenia (8.3%, 1/12), and leukopenia (8.3%, 1/12) were less altered in patients with coinfection (Fig 5).

### Treatments and treatment outcomes

Two studies [10,13] reported the treatment of patients with COVID-19. The study by Matangila *et al*. [10] conducted in Democratic Republic of Congo used hydroxychloroquine or chloroquine phosphate (92%), whereas the study by Mahajan *et al*. [13] conducted in India used hydroxychloroquine (53.9%) to treat patients with COVID-19.

Of the 12 cases of coinfection described in seven case reports and series [14–20], 5 of the patients involved were co-infected with *P*. *falciparum* and received oral artemether and lumefantrine; three were co-infected with *P*. *vivax* or *P*. *ovale* and received oral chloroquine; and one was co-infected with *P*. *vivax* and received artemether-lumefantrine, which was later changed to intravenous artesunate. The remaining three patients were co-infected with *P*. *vivax* (2 patients) and *P*. *falciparum* (1 patient) and received intravenous artesunate followed by oral artemether and lumefantrine. Of the 12 patients treated with anti-malarial drugs, five responded well, five showed uncomplicated signs or symptoms, including fever, abdominal pain, rigor, vomiting, nausea, blurring of vision, and breathing difficulty. One patient experienced worsened clinical status after the administration of artesunate. Another patient was initially hospitalized for COVID-19 treatment and then received anti-malarial treatment three weeks later because of failure to diagnose malaria during the first hospitalization.

## Discussion

COVID-19 was identified in December 2019 in Wuhan city of China and is currently affecting 146 countries worldwide [24]. Most of the evidence showed that COVID-19 is less pathogenic in children. However, severe manifestation and higher mortality rates were frequently observed in patients aged 65 years and above, and men had a higher risk of death than women [25–27]. While older individuals are at risk of developing a severe COVID-19 infection, children aged less than five are at risk of developing severe malaria [28–30]. For the age of patients with coinfection, it was reported in a wide range from 4−67 years [14–19]. Lockdown in countries during COVID-19 pandemic limited the access to health care facilities, suspended malaria chemoprevention, and suspended distribution of long-lasting insecticide-treated bed nets [7], which in turn caused the malaria outbreaks leading to deaths in malaria-endemic areas, such as Africa; therefore, continuing attention to malaria during COVID-19 pandemic by including both COVID-19 and malaria diagnoses in cases of fever, are needed [31].

Our analysis of the prevalence of *Plasmodium* spp. infection and characteristics of COVID-19 in patients co-infected with malaria and COVID-19 shows a high level (11%) of pooled prevalence of *Plasmodium* spp. infection. However, the prevalence of coinfection appears heterogeneous, and the lowest level of prevalence of *Plasmodium* spp. infection among COVID-19 patients (1%) is similar to that reported by Matangila *et al*. [10]. A low prevalence of coinfection can be explained by the low prevalence of malaria in the area investigated, with nearly half of patients receiving anti-malarial drugs before hospitalization [10]. The study by Mahajan *et al*. [13] has also demonstrated a low level of coinfection prevalence in India (5%), suggesting that COVID-19 and malaria coinfection may enhance recovery from COVID-19, with virus clearance being achieved by the glycosylphosphatidylinositol (GPI) antibodies (immunoglobulin G) against plasmodium-specific antigens cross-reacting with SARS-CoV-2 antibodies [13,23], or population exposed to malaria exhibits a higher prevalence of the naturally selected ACE2 rs2106806 TT/T genotype, which leads to ACE2 down-regulation, which in turn reduces the chances of SARS-CoV-2 entry into lung epithelial cells [2]. Nevertheless, although three studies reported a low prevalence of *Plasmodium* spp. infection among COVID-19 cases, two studies reported high prevalence levels (63%–100%) in Nigeria [11,12]. Muhammad *et al*. [11] reported 63% of malaria infection among COVID-19 cases. They suggested that increased levels of 8-iso-PGF2α in patients with coinfection are associated with deterioration of the patients’ condition because of a high oxidative stress-induced pro-inflammatory response against SARS-COV-2 infection [11]. Meanwhile, Onosakponome *et al*. [12] reported a 100% rate of *Plasmodium* spp. infection among COVID-19 cases, and that co-infected males tend to exhibit more symptoms than co-infected females. The prevalence of malaria and COVID-19 in Africa or WHO South-East Asia where malaria is endemic may be underreported or understudied because some patients with malaria may be asymptomatic. Some patients, particularly young ones, with COVID-19 may be asymptomatic. It is unlikely that these patients will be tested for either asymptomatic malaria or COVID-19. Therefore, the actual pooled prevalence of malaria and COVID-19 may be higher than 11%. Additionally, the limited testing capacity for conducting a confirmatory test for COVID-19 in Africa, RT-PCR which is expensive, multi-step processes, limiting the capacity for mass testing, shortages of testing kits, and requires well-trained healthcare staff, would lead to underreported or understudied COVID-19 in Africa [32–34]. Another explanation for the low prevalence of coinfection in urban settings was the low prevalence of malaria [35] and high prevalence of COVID-19 is high in these areas. Additionally, malaria is prevalent among children 5 years, and below, however, COVID-19 prevalence is low in this population. Therefore, these factors could be some explanation for the low prevalence of malaria and COVID-19 coinfection.

Our analysis shows that most patients with coinfection were symptomatic at presentation [10,12,13]. Also, an analysis of 12 co-infected individuals indicates that 58.3% of such individuals presented with fever and non-specific signs or symptoms. The clinical presentation of patients with COVID-19 ranged from asymptomatic to mild (e.g., fever, headache, cough, fatigue, myalgia, nausea, and vomiting) and severe (e.g., ARDS, shock, and multi-organ failures) symptoms [36]. These symptoms overlap with those produced by malaria infection, especially fever, which may be misdiagnosed as COVID-19 and vice versa. Furthermore, severe COVID-19 symptoms such as ARDS, shock, and multi-organ failures are severe complications of patients with severe malaria [37].

Our analysis indicates that most patients with coinfection have low or moderate parasitemia at presentation [10,12,15,18]. One exception was a patient with coinfection who showed jaundice, high parasitemia (119,902 parasites/μL), and disseminated intravascular coagulation (DIC), which were later treated following the WHO guidelines for treating severe malaria [16]. The cause of severe malaria in this patient with coinfection was the initial misdiagnosis of the case as dengue fever, pancreatitis, and pneumonia [16]. Jaundice with high levels of parasitemia occurred in high proportions of patients with severe malaria caused by *P*. *knowlesi* (42%) [38], *P*. *vivax* (18%) [39], *P*. *ovale* (15%) [40], and less commonly in patients with *P*. *falciparum* infection (1.18%) [40]. Therefore, in areas where *P*. *falciparum* infections are endemic, patients with fever, jaundice, high parasitemia level, and DIC should be monitored for COVID-19 and *P*. *falciparum* coinfection.

For the difference in laboratory alterations between COVID-19 infected patients and co-infected individuals, the previous study showed that most of the COVID–19 monoinfected patients had normal leukocyte count (72.1%), elevated CRP (100%), elevated procalcitonin (74%), higher D-dimer (67%), elevated AST (66%), elevated ALT (37%), nearly normal urea and creatinine [10], whereas patients with coinfection[14–20] demonstrated thrombocytopenia (41.7%), lymphopenia (33.3%), elevated bilirubin (33.3%), elevated D-dimer (25%), elevated ferritin (25%), elevated CRP (25%), and mild anemia (16.7%). Therefore, it is likely that thrombocytopenia and lymphopenia might be possible makers for co-infected individuals. Additionally, COVID-19 infected patients have a higher proportion of laboratory alterations than those of co-infected individuals such as CRP, D-dimer, AST, and ALT. Nevertheless, thrombocytopenia, lymphopenia, mild anemia, elevated bilirubin, elevated D-dimer, and elevated ferritin were observed in *Plasmodium* monoinfected individuals [41–44]. Therefore, it is not clear whether laboratory parameters could be semi-diagnostic parameters for COVID-19 and *Plasmodium* co-infected individuals.

Thrombocytopenia occurs in approximately 80% of patients infected with either severe or uncomplicated malaria [45]. Lymphopenia and high neutrophil per lymphocyte ratios occur in malaria patients [41], and previous studies have also frequently noted lymphopenia in severe COVID-19 patients [46,47]. Such cases of lymphopenia indicate low T and B cell counts [48–50], which may be caused by coronaviruses affecting bone marrow precursors directly, resulting in increased auto-immune response against blood cell precursors [51,52]. Although lymphopenia is observed in severe patient with COVID-19, most of the COVID–19 monoinfected individuals with mild diseases have normal leukocyte count (72.1%) [10].

Platelets are another hematological parameter affected by coronaviruses infecting the bone marrow; decreased platelet levels result in thrombocytopenia. Platelet growth inhibition, platelet apoptosis, increased platelet consumption, or decreased production of platelets have all been suggested as mechanisms of thrombocytopenia in patients with COVID-19 [52]. Although thrombocytopenia may be a predictor of the severity and mortality of COVID-19, this condition is not associated with malaria severity, and it is not a criterion for severe malaria [53]. A previous study showed that patients with severe COVID-19 have higher bilirubin levels than those with non-severe symptoms [54]. This was supported by another meta-analysis that reported a higher risk of liver injury, as indicated by elevated levels of ALT, AST, and hyperbilirubinemia, in patients with severe COVID-19 who are critically ill [55].

Analysis of data from 12 patients with coinfection shows that four (3 with *P*. *vivax*, one with *P*. *falciparum*) received intravenous artesunate. These patients experienced vomiting [18], lung complications with progressive exertional breathlessness [20], jaundice with a high level of parasitemia [16], and post-datism [17]. A more serious outcome was reported in a pregnant woman whose fetus died, resulting in an abortion. This outcome was due to an initial misdiagnosis resulting in delayed management for malaria [17]. The misdiagnosis of malaria at initial presentation has also been reported in pregnant women co-infected with *P*. *ovale*; they were eventually treated with oral chloroquine during their second hospitalization after COVID-19 treatment [19].

The present study has the following limitations. First, since this is a meta-analysis, sample size is not pre-determined, but the numbers of patients are small due to the low number of qualifying studies conducted. Second, few countries are represented, especially given the expanse of malaria and a COVID-19 pandemic, which makes it difficult to derive conclusions about coinfections yet. Third, on limited amounts of clinical data, laboratory test results, treatments, and treatment outcomes between patients with coinfection, COVID-19 only, or malaria only could not be compared, and further studies need to be done for this research gap. Therefore, the meta-analysis was limited by the data generated in the included studies. Fourth, our analysis is based on a limited number of case reports and series involving COVID-19 and malaria coinfection. This may be due to underreporting or a lower prevalence of COVID-19 in malaria-endemic areas such as Africa. Underreported COVID-19 and malaria coinfection might be caused by patients who received anti-malarial treatment before their hospitalization with COVID-19 [10]. Fifth, there was another confounder in interpreting coinfections with COVID and *P*. *vivax*. While chloroquine or hydroxychloroquine has not been shown to be effective for COVID-19 [56], it is effective for *P*. *vivax* malaria in countries where *P*. *vivax* are chloroquine-sensitive [37]. Therefore, the included study that was conducted in India [13], could have a bias in interpreting symptoms or laboratory parameters for malaria. Nevertheless, the capacity to identify COVID-19 and malaria coinfections early must be strengthened in malaria-endemic areas. We suggest that healthcare centers may prevent the development of severe diseases by testing all febrile or COVID-19-symptomatic patients for malaria infection using either microscopy or rapid diagnostic tests. These tests should be performed before the patients return home. Further studies are required to address the impact of malaria infection on COVID-19 individuals and investigate the prevalence of malaria and COVID-19 coinfection in asymptomatic patients. These studies will help develop prompt management strategies in all coinfected individuals, thus reducing poor patient outcomes during the COVID-19 pandemic.

## Supporting information

S1 PRISMA Checklist (DOC)

S1 Table Search terms. (DOCX)

S2 Table Quality of the included studies. (DOCX)

S1 Data Characteristics of co-infected cases from case reports and series. (XLSX)
