# Supplementary material for: Prevalence and characteristics of malaria among COVID-19 individuals: A systematic review, meta-analysis, and analysis of case reports
Source: PLoS Negl Trop Dis. 2021 Oct 1;15(10):e0009766. doi: 10.1371/journal.pntd.0009766 (PMC8486116; doi:10.1371/journal.pntd.0009766)
Supplement: S1 Table — (DOCX) [file pntd.0009766.s002.docx]

**Prevalence and characteristics of malaria among COVID-19 individuals: a systematic review, meta-analysis, and analysis of case reports**

Polrat Wilairatana^1^, Frederick Ramirez Masangkay^2^, Kwuntida Uthaisar Kotepui^3^, Giovanni De Jesus Milanez^4^, Manas Kotepui^3*^

^1^Department of Clinical Tropical Medicine, Faculty of Tropical Medicine, Mahidol University, Bangkok, Thailand

^2^Department of Medical Technology, Institute of Arts and Sciences, Far Eastern University-Manila, Manila, Philippines

^3^Medical Technology, School of Allied Health Sciences, Walailak University, Tha Sala, Nakhon Si Thammarat, Thailand

^4^Department of Medical Technology, Faculty of Pharmacy, University of Santo Tomas, Manila, Philippines.

**^*^Corresponding author**

Email: manas.ko@wu.ac.th, Tel.: +66954392469

Polrat Wilairatana; [polrat.wil@mahidol.ac.th](mailto:polrat.wil@mahidol.ac.th)

Frederick Ramirez Masangkay; [frederick_masangkay2002@yahoo.com](mailto:frederick_masangkay2002@yahoo.com)

Kwuntida Uthaisar Kotepui; [kwuntida.ut@wu.ac.th](mailto:kwuntida.ut@wu.ac.th)

Giovanni De Jesus Milanez; gmilanez81@gmail.com

**Table S1. Search terms**

| **Databases** | **Search terms** | **Search date** |
| --- | --- | --- |
| MEDLINE (PubMed) | (“COVID-19” OR “2019-nCoV” OR “2019 nCoV Infection” OR “2019-nCoV” OR “Coronavirus Disease-19” OR “Coronavirus Disease 19” OR “2019 Novel Coronavirus” OR COVID19 OR “COVID 19” OR “COVID-19” OR “SARS Coronavirus 2” OR “SARS-CoV-2” OR “SARS-CoV-2”) AND (malaria OR Plasmodium)  Search option: All fields  Search results: 368 | 27 March 2021 |
| Scopus | ((COVID-19) OR (2019-nCoV) OR (2019 nCoV Infection) OR (2019-nCoV) OR (Coronavirus Disease-19) OR (Coronavirus Disease 19) OR (2019 Novel Coronavirus) OR COVID19 OR (COVID 19) OR (COVID-19) OR (SARS Coronavirus 2) OR (SARS-CoV-2) OR (SARS-CoV-2)) AND (malaria OR Plasmodium)  Search option: title, abstract, keywords  Search results: 501 | 27 March 2021 |
| ISI Web of Science | (“COVID-19” OR “2019-nCoV” OR “2019 nCoV Infection” OR “2019-nCoV” OR “Coronavirus Disease-19” OR “Coronavirus Disease 19” OR “2019 Novel Coronavirus” OR COVID19 OR “COVID 19” OR “COVID-19” OR “SARS Coronavirus 2” OR “SARS-CoV-2” OR “SARS-CoV-2”) AND (malaria OR Plasmodium)  Search option: All fields  Search results: 338 | 27 March 2021 |
