# Supplementary material for: Prevalence and characteristics of malaria among COVID-19 individuals: A systematic review, meta-analysis, and analysis of case reports
Source: PLoS Negl Trop Dis. 2021 Oct 1;15(10):e0009766. doi: 10.1371/journal.pntd.0009766 (PMC8486116; doi:10.1371/journal.pntd.0009766)
Supplement: S2 Table — (DOCX) [file pntd.0009766.s003.docx]

**Prevalence and characteristics of malaria among COVID-19 individuals: a systematic review, meta-analysis, and analysis of case reports**

Polrat Wilairatana^1^, Frederick Ramirez Masangkay^2^, Kwuntida Uthaisar Kotepui^3^, Giovanni De Jesus Milanez^4^, Manas Kotepui^3*^

^1^Department of Clinical Tropical Medicine, Faculty of Tropical Medicine, Mahidol University, Bangkok, Thailand

^2^Department of Medical Technology, Institute of Arts and Sciences, Far Eastern University-Manila, Manila, Philippines

^3^Medical Technology, School of Allied Health Sciences, Walailak University, Tha Sala, Nakhon Si Thammarat, Thailand

^4^Department of Medical Technology, Faculty of Pharmacy, University of Santo Tomas, Manila, Philippines.

**^*^Corresponding author**

Email: manas.ko@wu.ac.th, Tel.: +66954392469

Polrat Wilairatana; [polrat.wil@mahidol.ac.th](mailto:polrat.wil@mahidol.ac.th)

Frederick Ramirez Masangkay; [frederick_masangkay2002@yahoo.com](mailto:frederick_masangkay2002@yahoo.com)

Kwuntida Uthaisar Kotepui; [kwuntida.ut@wu.ac.th](mailto:kwuntida.ut@wu.ac.th)

Giovanni De Jesus Milanez; gmilanez81@gmail.com

**Table S2.** Quality of the included studies

| **No.** | **Authors** | **Eligibility criteria** | **Study subjects and the setting** | **Exposure measured in a valid and reliable way 'gold standard'** | **A specified diagnosis or definition** | **Confounding factors** | **Dealing with confounding factors** | **Outcomes measured in a valid and reliable way** | **Appropriate statistical analysis** | **Scores (8)** | **Quality (high, moderate, low)** |
| --- | --- | --- | --- | --- | --- | --- | --- | --- | --- | --- | --- |
| 1 | Amoo et al., 2020 [1] | Yes | Yes | Yes | Yes | No | NA | Yes | Yes | 7 | High |
| 2 | Matangila et al., 2020 [2] | Yes | Yes | Yes | Yes | No | NA | Yes | Yes | 7 | High |
| 3 | Muhammad et al., 2020 [3] | Yes | No | Yes | Yes | No | NA | Yes | Yes | 6 | Moderate |
| 4 | Onosakponome et al., 2020 [4] | Yes | Yes | Yes | Yes | No | NA | Yes | Yes | 7 | High |
| 5 | Mahajan et al., 2021 [5] | Yes | Yes | Yes | Yes | No | NA | Yes | Yes | 7 | High |

🟑A star rating, NA: Not assessed

**References**

1. Amoo OS, Odubela A, Okwuraiwe AP, Onwuamah CK, Shaibu JO, Ige F, Owaneze K, Agboola HO, Kareithi DN, Onuigbo TI, Ikemefuna AS. COVID-19 spread patterns is unrelated to malaria co-infections in Lagos, Nigeria. Adv Infect Dis. 2020;10: 200-215.
2. Matangila JR, Nyembu RK, Telo GM, Ngoy CD, Sakobo TM, Massolo JM, et al. Clinical characteristics of COVID-19 patients hospitalized at Clinique Ngaliema, a public hospital in Kinshasa, in the Democratic Republic of Congo: A retrospective cohort study. PLoS One. 2020;15: 15.
3. Muhammad Y, Aminu YK, Ahmad AE, Iliya S, Muhd N, Yahaya M, et al. An elevated 8-isoprostaglandin F2 alpha (8-iso-PGF2α) in COVID-19 subjects co-infected with malaria. Pan Afr Med J. 2020;37: 1-10.
4. Onosakponome EO, Wogu MN. The Role of Sex in Malaria-COVID19 Coinfection and some associated factors in Rivers State, Nigeria. J Parasitol Res. 2020;2020: 4.
5. Mahajan NN, Gajbhiye RK, Bahirat S, Lokhande PD, Mathe A, Rathi S, et al. Co-infection of malaria and early clearance of SARS-CoV-2 in healthcare workers. J Med Virol. 2021;93: 2431-2438.
